# Management of Acute Exacerbation of Idiopathic Pulmonary Fibrosis in Specialised and Non-specialised ILD Centres Around the World

**DOI:** 10.3389/fmed.2021.699644

**Published:** 2021-09-27

**Authors:** Markus Polke, Yasuhiro Kondoh, Marlies Wijsenbeek, Vincent Cottin, Simon L. F. Walsh, Harold R. Collard, Nazia Chaudhuri, Sergey Avdeev, Jürgen Behr, Gregory Calligaro, Tamera J. Corte, Kevin Flaherty, Manuela Funke-Chambour, Martin Kolb, Johannes Krisam, Toby M. Maher, Maria Molina Molina, Antonio Morais, Catharina C. Moor, Julie Morisset, Carlos Pereira, Silvia Quadrelli, Moises Selman, Argyrios Tzouvelekis, Claudia Valenzuela, Carlo Vancheri, Vanesa Vicens-Zygmunt, Julia Wälscher, Wim Wuyts, Elisabeth Bendstrup, Michael Kreuter

**Affiliations:** ^1^Center for Interstitial and Rare Lung Diseases, Pneumology, Thoraxklinik, University of Heidelberg, Heidelberg, Germany; ^2^Department of Respiratory Medicine and Allergy, Tosei General Hospital, Seto, Japan; ^3^Department of Respiratory Medicine, Centre for Interstitial Lung Diseases and Sarcoidosis, Erasmus University Medical Centre, Rotterdam, Netherlands; ^4^National Coordinating Reference Centre for Rare Pulmonary Diseases, Louis Pradel Hospital, Hospices Civils de Lyon, University Claude Bernard Lyon 1, Lyon, France; ^5^Imperial College, National Heart and Lung Institute, London, United Kingdom; ^6^Department of Medicine, University of California, San Francisco, San Francisco, CA, United States; ^7^North West Interstitial Lung Disease Unit, Manchester University NHS Foundation Trust, Wythenshawe, Manchester, United Kingdom; ^8^Sechenov First Moscow State Medical University, Moscow, Russia; ^9^Medizinische Klinik und Poliklinik V, LMU Klinikum, University of Munich, Munich, Germany; ^10^German Center for Lung Research (DZL), Marburg, Germany; ^11^Division of Pulmonology, Department of Medicine, University of Cape Town, Cape Town, South Africa; ^12^Royal Prince Alfred Hospital, University of Sydney, Sydney, NSW, Australia; ^13^Department of Medicine, University of Michigan, Ann Arbor, MI, United States; ^14^Department of Pulmonary Medicine, Inselspital, Bern University Hospital, University of Bern, Bern, Switzerland; ^15^Department of Medicine, Firestone Institute for Respiratory Health, Research Institute at St Joseph's Healthcare, McMaster University, Hamilton, ON, Canada; ^16^Institute of Medical Biometry and Informatics, University of Heidelberg, Heidelberg, Germany; ^17^Hastings Centre for Pulmonary Research and Division of Pulmonary, Critical Care, and Sleep Medicine, Keck School of Medicine, University of Southern California, Los Angeles, CA, United States; ^18^Interstitial Lung Disease Unit, Imperial College London, National Heart and Lung Institute, Royal Brompton and Harefield NHS Foundation Trust, London, United Kingdom; ^19^Institut d'Investigació Biomèdica de Bellvitge (IDIBELL), University Hospital of Bellvitge, L'Hospitalet de Llobregat, Barcelona, Spain; ^20^Centro de Investigación Biomédica en Red Enfermedades Respiratorias (CIBERES), Madrid, Spain; ^21^Department of Pneumology, Faculdade de Medicina, Centro Hospitalar São João, Universidade do Porto, Porto, Portugal; ^22^Département de Médecine, Centre Hospitalier de l'Université de Montréal, Montréal, QC, Canada; ^23^Lung Disease Department, Paulista School of Medicine, Federal University of São Paulo, São Paulo, Brazil; ^24^Hospital Británico, Buenos Aires, Argentina; ^25^Sanatorio Güemes, Buenos Aires, Argentina; ^26^Instituto Nacional de Enfermedades Respiratorias Ismael Cosío Villegas, Mexico City, Mexico; ^27^Department of First Academic Respiratory, Sotiria General Hospital for Thoracic Diseases, University of Athens, Athens, Greece; ^28^ILD Unit, Pulmonology Department Hospital Universitario de La Princesa, Universidad Autonoma de Madrid, Madrid, Spain; ^29^Regional Referral Centre for Rare Lung Diseases, A.O.U. Policlinico-Vittorio Emanuele, University of Catania, Catania, Italy; ^30^Unit of Interstitial Lung Diseases, Department of Pneumology, Pneumology Research Group, IDIBELL, L'Hospitalet de Llobregat, University Hospital of Bellvitge, Barcelona, Spain; ^31^Department of Pulmonary Medicine, Centre for Interstitial and Rare Lung Diseases, Ruhrlandklinik University Hospital Essen, Essen, Germany; ^32^Unit for Interstitial Lung Diseases, Department of Respiratory Diseases, University Hospitals Leuven, Leuven, Belgium; ^33^Department of Respiratory Diseases and Allergy, Aarhus University Hospital, Aarhus C, Denmark

**Keywords:** idiopathic pulmonary fibrosis, acute exacerbation, questionnaire, pulmonologists, specialised ILD centres, non-specialised ILD centres

## Abstract

**Background:** Acute exacerbation of idiopathic pulmonary fibrosis (AE-IPF) is a severe complication associated with a high mortality. However, evidence and guidance on management is sparse. The aim of this international survey was to assess differences in prevention, diagnostic and treatment strategies for AE-IPF in specialised and non-specialised ILD centres worldwide.

**Material and Methods:** Pulmonologists working in specialised and non-specialised ILD centres were invited to participate in a survey designed by an international expert panel. Responses were evaluated in respect to the physicians' institutions.

**Results:** Three hundred and two (65%) of the respondents worked in a specialised ILD centre, 134 (29%) in a non-specialised pulmonology centre. Similarities were frequent with regards to diagnostic methods including radiology and screening for infection, treatment with corticosteroids, use of high-flow oxygen and non-invasive ventilation in critical ill patients and palliative strategies. However, differences were significant in terms of the use of KL-6 and pathogen testing in urine, treatments with cyclosporine and recombinant thrombomodulin, extracorporeal membrane oxygenation in critical ill patients as well as antacid medication and anaesthesia measures as preventive methods.

**Conclusion:** Despite the absence of recommendations, approaches to the prevention, diagnosis and treatment of AE-IPF are comparable in specialised and non-specialised ILD centres, yet certain differences in the managements of AE-IPF exist. Clinical trials and guidelines are needed to improve patient care and prognosis in AE-IPF.

## Introduction

Idiopathic pulmonary fibrosis (IPF) is a chronic and progressive fibrosing interstitial lung disease associated with a poor prognosis with a five-year survival rate of 20–40% and a median survival time of 2–5 years (1, 2). An acute exacerbation of IPF contributes to the dismal prognosis and disease progression and is defined as an acute, clinically significant respiratory deterioration characterised by evidence of new widespread alveolar abnormality in patients with IPF and after the exclusion of cardiac failure or fluid overload (3). The annual incidence is up to 20%, depending on the population analysed (4, 5). AE-IPF is associated with a median survival of ~3–4 months (6, 7) and may account for more than 40% of all death in patients with IPF (8). The aetiology is still obscure, but AE-IPF might be triggered e.g. by infection or procedures/operation, or may occur idiopathic (6). Evidence on prevention, diagnosis and therapy of AE-IPF is sparse and no international guidelines exist (3, 9). Accordingly, there is a huge variability with regards to preventive, diagnostic and therapeutic approaches worldwide (10). It is unknown whether these different strategies are partially explainable by differences between specialised and non-specialised ILD centres. Therefore, this study aimed to compare preventive, diagnostic and therapeutic strategies for AE-IPF between specialised and non-specialised ILD centres.

## Materials and Methods

### Questionnaire and Participating Physicians

As described previously (10), as a first step we conducted a literature research on diagnostics, therapy, prevention and management of AE-IPF to identify items to be included in this survey. Then, an expert panel was created, comprising pulmonologists with expertise in the diagnosis and management of ILD working in specialist ILD centres and a track record of publication in this field, to participate in an email-based interview to structure the survey. The final questionnaire consisted of 20 questions regarding diagnosis, treatment and prevention of AE-IPF and suggested future perspectives in AE-IPF research (10). To identify working place (specialised and non-specialised ILD centres), country of origin, number of patients with IPF under care, and estimated number of AE-IPF seen, additional questions were included into this survey. From July 1 2017 to November 30 2017 pulmonologists worldwide with interest in ILD were identified, including the European Respiratory Society assembly on Diffuse Parenchymal Lung Disease, the American Thoracic Society assembly on Clinical Problems, the Japanese Respiratory Society assembly on Diffuse Parenchymal Lung Disease and participants of the IPF Project Consortium (www.theipfproject.com) (11). Nationality, academic status (working at a university hospital or not) or subspecialist interests within respiratory medicine did not influence inclusion eligibility. The questionnaire was provided by the online survey tool SurveyMonkey® from December 2017 to April 2018.

### Statistical Analysis

For questions with categorical answers, absolute and relative frequencies were calculated and chi-squared tests were used to assess differences between specialised and non-specialised ILD centres. For questions with answers on a continuous scale, median, first and third quartile, minimum and maximum were determined and differences between continents were assessed using Kruskal–Wallis tests. Due to the exploratory nature of this survey, all resulting *p*-values are solely to be interpreted descriptively and no adjustment for multiple testing was conducted. *p*-values smaller than 0.05 were regarded as statistically significant. All analyses were conducted using R v.3.4.2 (http://r-project.org).

## Results

### Participants

**Figure 1:**
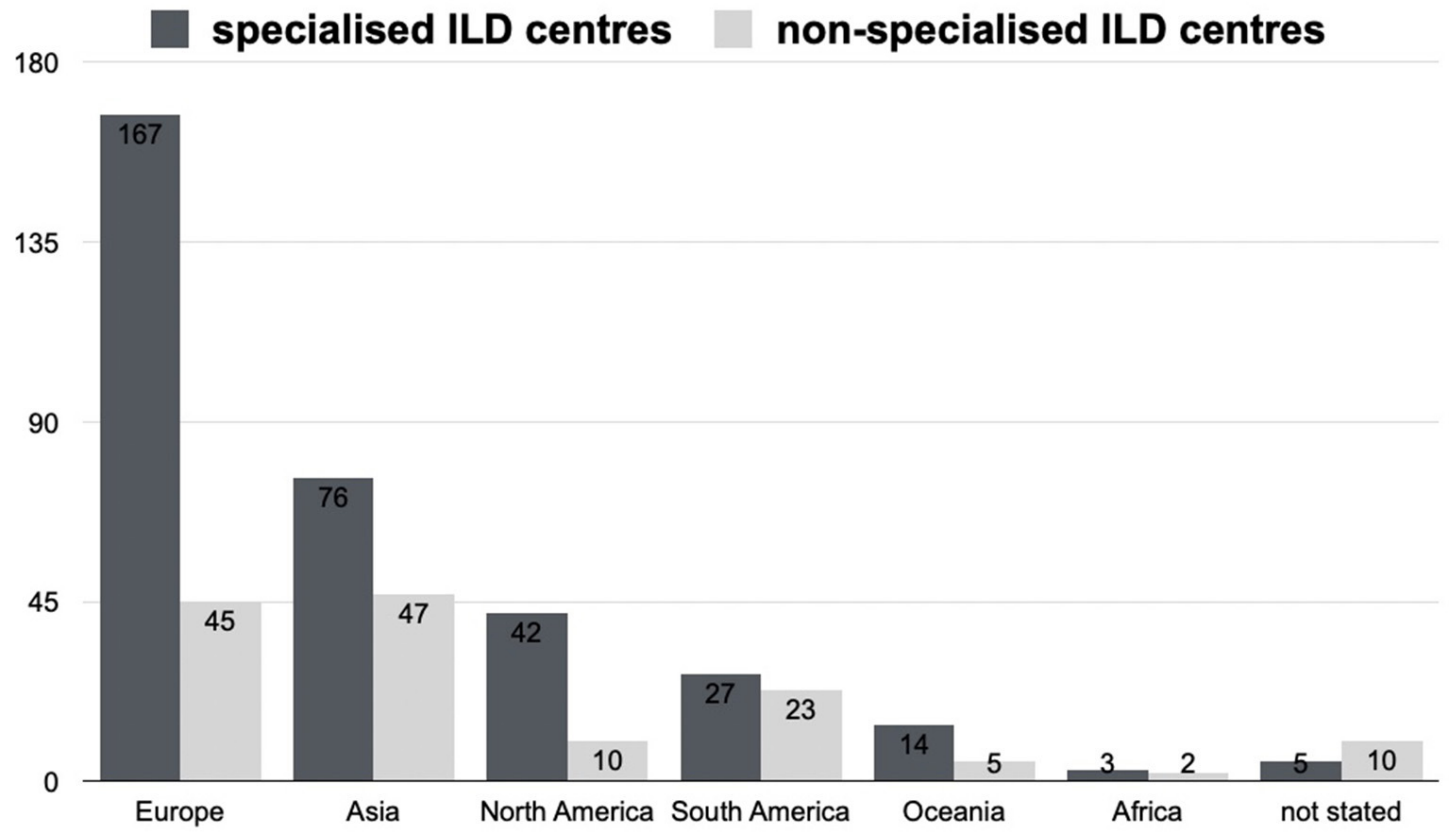
Number of participants in specialised and non-specialised ILD centres from different continents.

Overall, 509 pulmonologists from 66 countries responded. Three hundred and thirty four (65.9%) of the participants worked in a specialised ILD centre, 142 (28.0%) in non-specialised ILD centres i.e. in a general pulmonology department, 4 (0.8%) on an intensive care unit and 27 (5.3%) did not indicate their institution. Physicians working on an intensive care unit or who did not indicate their institution were excluded from the analysis. A total of 436 pulmonologists working in a specialised or non-specialised ILD centre were included in this analysis. On average 331 cases of AE-IPF were seen in specialised ILD centres and 139 in non-specialised ILD centres. Figure 1 shows the place of work (continent) of the respondents in specialised and non-specialised ILD centres.

### Diagnostic Procedures for AE-IPF

**Figure 2:**
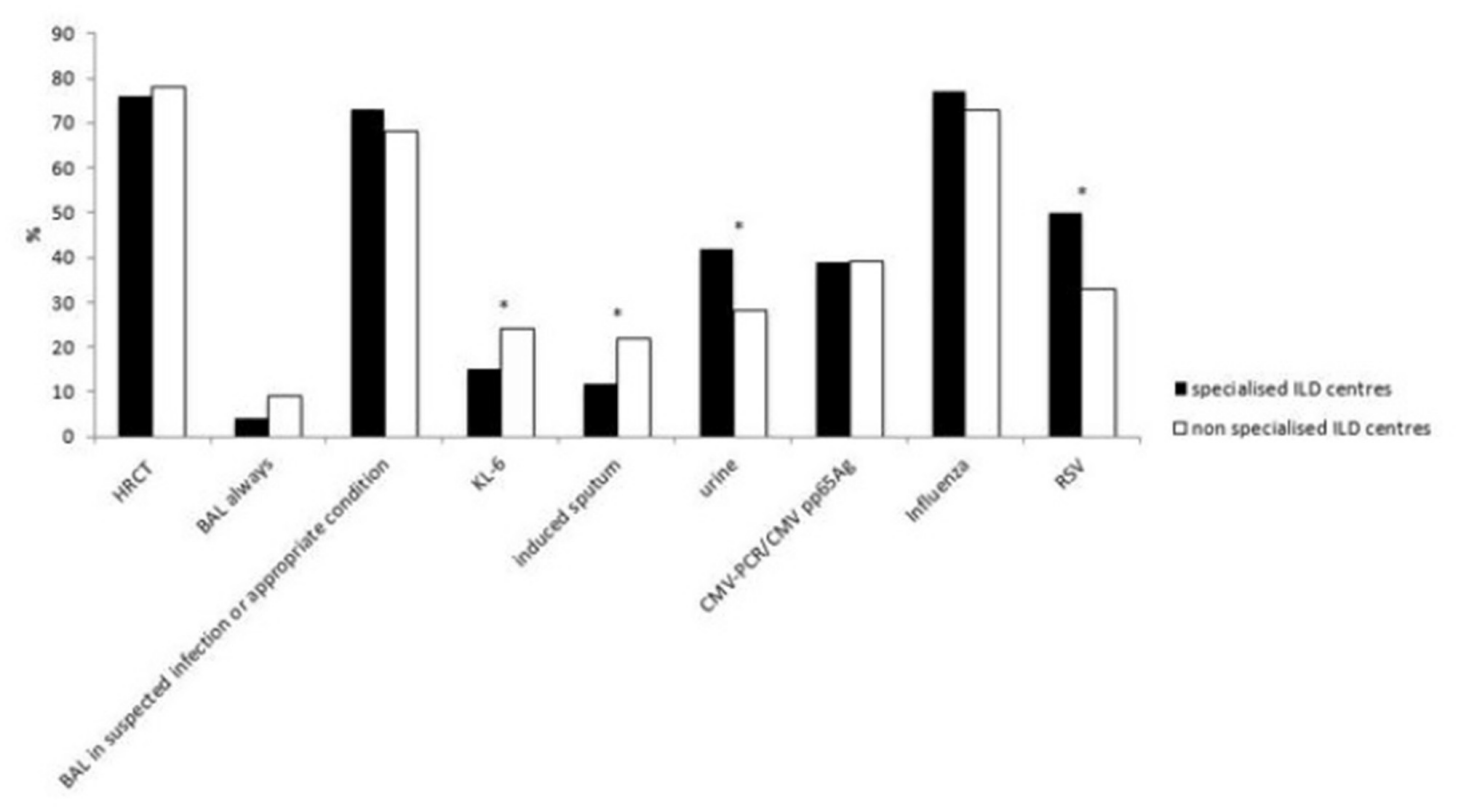
The main diagnostic procedures applied for AE-IPF in specialised and non-specialised ILD centres. Statistically significant differences are labelled with a ^*^ (p-value = <0.05).

Most diagnostic tools, including multi-slice thin-section computer tomography without contrast media (HRCT), CT with contrast media in the absence of clinical suspicion of pulmonary embolism, bronchoalveolar lavage (BAL), echocardiography, assessment for pathogens, NT-proBNP/BNP, D-Dimer and troponins were used similarly between specialised and non-specialised ILD centres. The main difference was the sampling of sputum for microbiology (induced sputum) which was more frequent in non-specialised ILD centres (22 vs. 12%, *p* = 0.0106). Conversely, pathogen testing in urine was performed significantly more often in specialised ILD centres than in non-specialised ILD centres (42 vs. 28%, *p* = 0.0068). 50% of specialised ILD centres screened for RSV (respiratory syncytial virus), compared to only 33% in non-specialised ILD centres (*p* = 0.0024). The use of biomarker KL-6 was higher in non-specialised ILD centres than in specialised ILD centres (24 vs. 15%, *p* = 0.0313). The most relevant diagnostic procedures applied for AE-IPF are shown in Figure 2. Other diagnostic procedures such as laboratory parameters or specific pathogens are shown in Supplementary 1.

### Treatment Approaches for AE-IPF

The majority of participating pulmonologists treated AE-IPF with methylprednisolone or equivalent with a dosage of 500–1,000 mg per day for 3 days followed by a slow tapering similarly in both types of institutions (63 vs. 66%).

Other immunosuppressive therapies such as cyclosporine, cyclophosphamide (i.v. bolus), tacrolimus and rituximab were rarely used in both specialised and non-specialised ILD centres, but cyclosporine was significantly more frequently used in non-specialised ILD centres (13 vs. 6%, *p* = 0.0288).

Other therapies such as polymyxin B hemoperfusion (7 vs. 10%), and plasmapheresis/plasma exchange (4 vs. 5%) were also less commonly used in specialised and non-specialised ILD centres. However, significantly more pulmonologists treated their patients with recombinant thrombomodulin in non-specialised ILD centres than in specialised ILD centres (15 vs. 8%, *p* = 0.0342).

**Figure 3:**
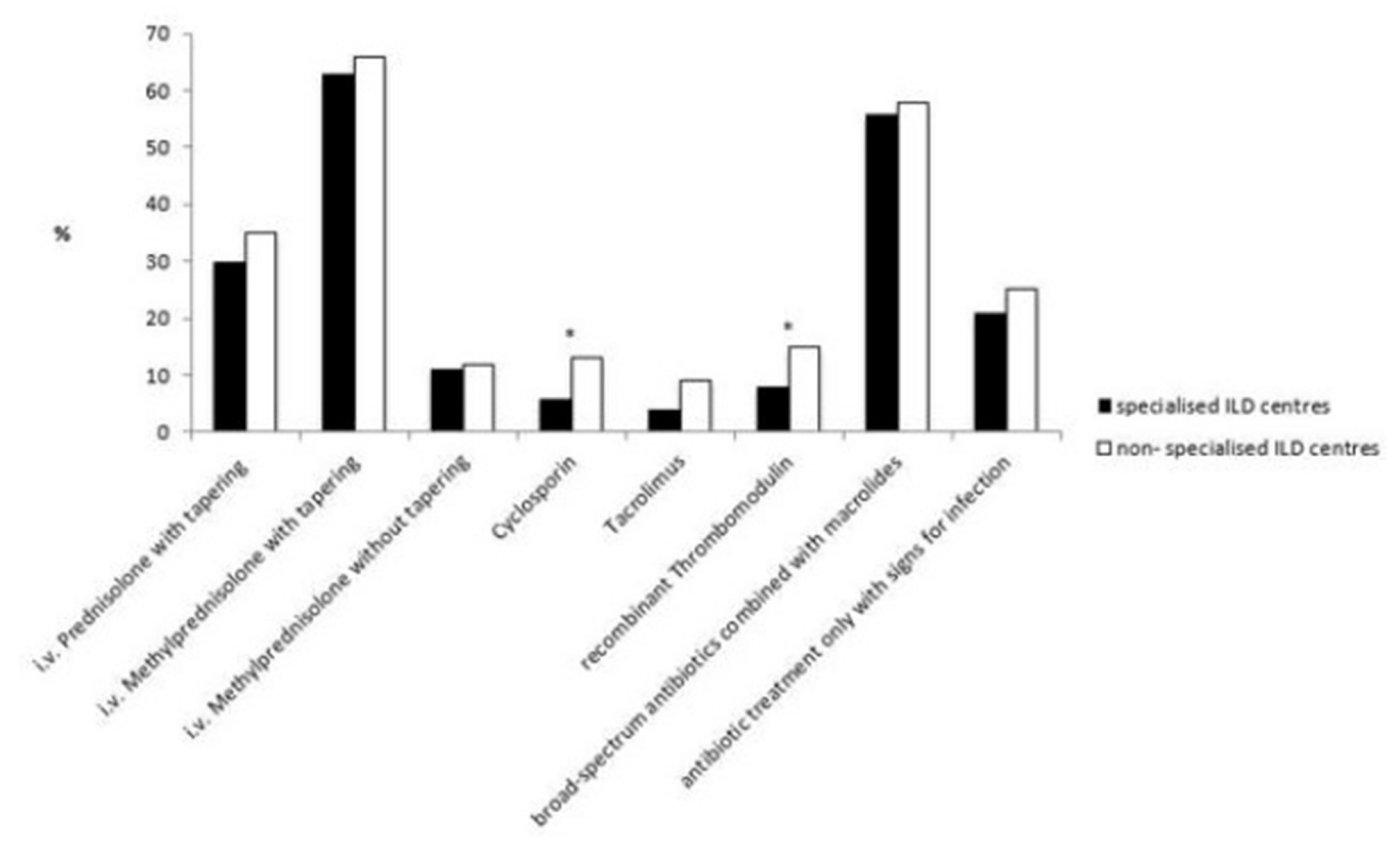
Different treatment strategies towards AE-IPF in specialised and non-specialised ILD centres. Statistically significant differences are labelled with a ^*^ (p-value = <0.05).

Differences between institutions in the use of treatment strategies are shown in Figure 3. *See also* Supplementary 1.

### Antifibrotic Therapy Management During AE-IPF

In patients without antifibrotic therapy, the majority of the survey participants see a reason to initiate an antifibrotic therapy in the event of an AE-IPF in specialised and non-specialised ILD centres (66 vs. 69%).

The choice of the antifibrotic drug did also not differ significantly between specialised and non-specialised centres (nintedanib 20 vs. 20%, pirfenidone 11 vs. 19%, no preference for specific anti-fibrotic drug 35 vs. 28%).

For patients already on antifibrotic therapy at the time of AE-IPF, there was no significant difference in the approach: 80% of respondents in specialised ILD centres would continue and 5% would discontinue antifibrotic therapy, compared to 70% continuing (*p* = 0.0513) and 7% discontinuing therapy (*p* = 0.5491) in non-specialised ILD centres. The dose was reduced by 1 % in specialised ILD centres compared to 5% in non-specialised ILD centres (*p* = 0.0301), 9% would switch to the alternative antifibrotic therapy in specialist ILD centres and similarly 10% would switch in non-specialised ILD centres (*p* = 0.999).

**Figure 4:**
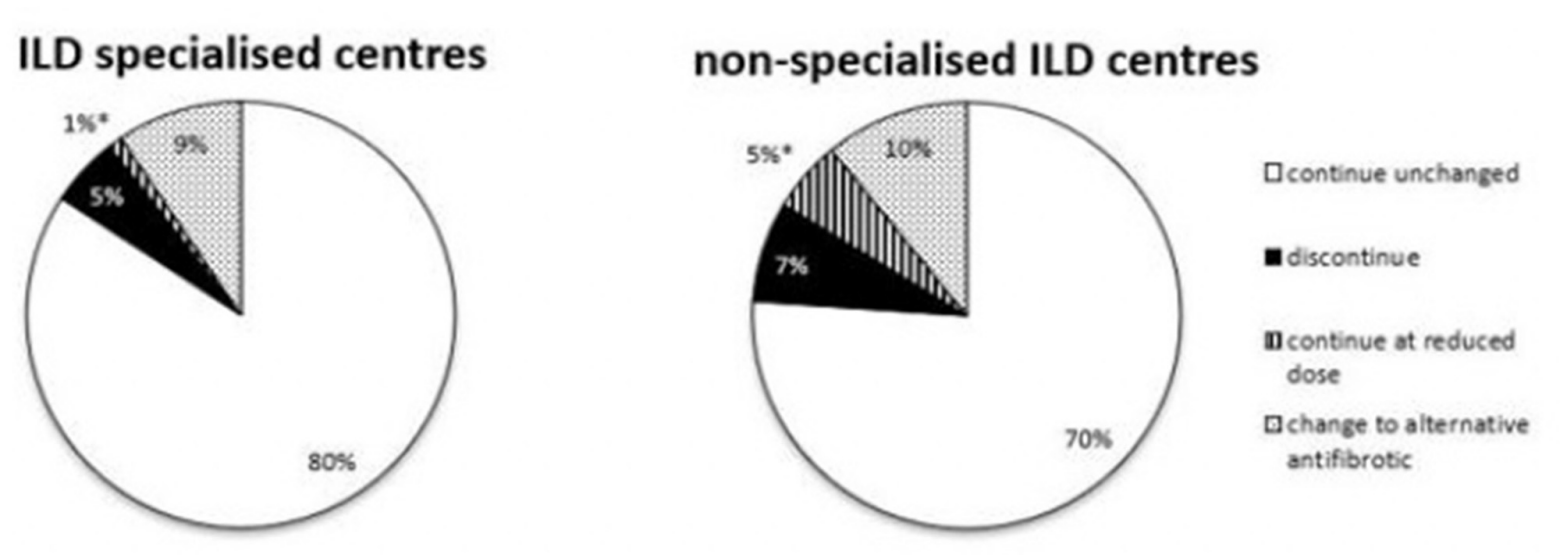
Different strategies in AE-IPF concerning an antifibrotic treatment in specialised and non-specialised ILD centres. Statistically significant differences are labelled with a ^*^ (p-value = <0.05).

Different strategies in this situation are also shown in Figure 4. For further strategies *see* Supplementary 1.

### Management of Pulmonary Hypertension (PH) During AE-IPF

In the case of suspected PH on clinical investigations (e.g. echocardiography, BNP, clinical signs) during an AE-IPF, significantly more physicians in specialised ILD centres would start diuretic therapy than in non-specialised ILD centre (54 vs. 41%, *p* = 0.0210). Only a minority in both institutions would perform right heart catheterization in AE-IPF in suspected PH (6 vs. 7%). Seven percentage would start a PH specific treatment after an established PH diagnosis in a specialised ILD centre, significantly more (14%) would do so in a non-specialised ILD centre (*p* = 0.0494). Only 3% of physicians in a specialised ILD centre and 1% in a non-specialised centre would start a PH specific treatment without a confident diagnosis. After stabilisation of AE-IPF, more than 50% of physicians would evaluate a PH specific treatment by subsequently performing right heart catheterization (56 vs. 55%). A quarter of all participating pulmonologists in specialised and non-specialised ILD centres saw no indication for a PH treatment during or after AE-IPF (Supplementary 1).

### Intensive Care Unit (ICU) and Palliative Care in AE-IPF

With regards to the care for critically ill patients with AE, there were no differences in specialised and non-specialised ILD centres for the use of high-flow oxygen (84 vs. 78%) and non-invasive ventilation (NIV) (72 vs. 77%). 9% of specialised and 11% of non-specialised ILD centres use invasive ventilation for all critical ill patients, whereas 48 vs. 39% respectively would only use invasive ventilation in patients suitable for lung transplantation (LTX) as a bridge to LTX or in very selected cases.

Significantly more physicians in specialised ILD centres offered extracorporeal membrane oxygenation (ECMO) to patients suitable for LTX as a bridge to LTX than in non-specialised ILD centres (49 vs. 36%, *p* = 0.0287).

Palliative care was considered similarly in both types of institutions (65 vs. 62%).

**Figure 5:**
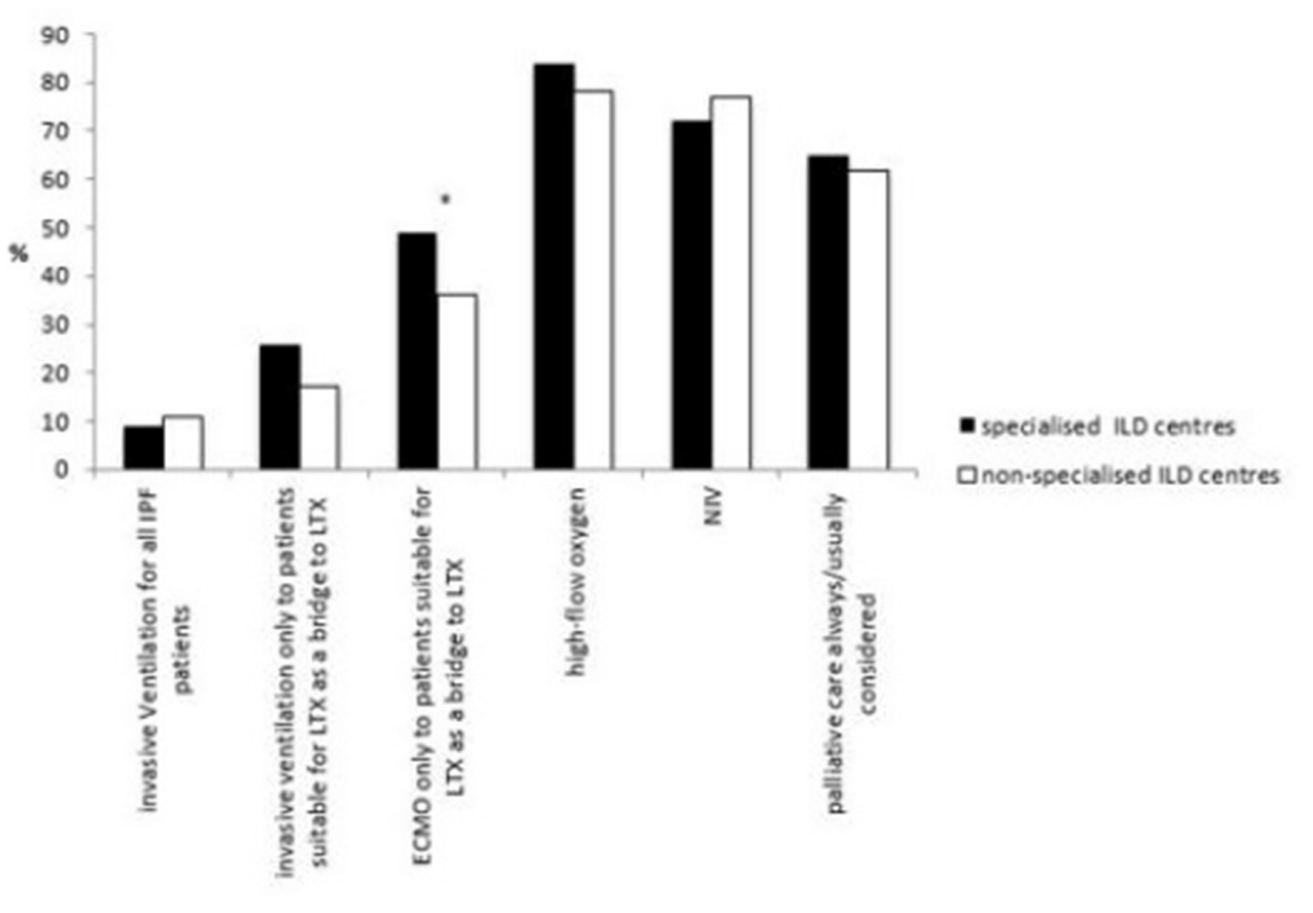
Different management strategies in critically ill patients with AE-IPF in specialised and non-specialised ILD centres. Statistically significant differences are labelled with a ^*^ (p-value = <0.05).

Institutional differences in these approaches are shown in Figure 5 (Supplementary 1).

### Preventive Strategies for AE-IPF

Measures aimed at preventing the occurrence of AE-IPF was equal amongst specialised and non-specialised ILD centres and included vaccinations, antifibrotic therapy and pulmonary rehabilitation or other forms of structured exercise therapy. Antacid drugs were prescribed significantly more in non-specialised ILD centres than in specialised ILD centres (61 vs. 50%, *p* = 0.0438) in all IPF patients. Long-term azithromycin, low dose steroids (≤10 mg) and anticoagulation (to prevent AE-IPF) were only used by a minority in both types of institutions.

In terms of planned surgical procedures, significantly more physicians in specialised ILD centres favoured preventive anaesthetic measures such as low tidal volume and avoidance of hyperoxygenation compared to physicians in non-specialised ILD centres (72 vs. 61%, p=0.0368).

**Figure 6:**
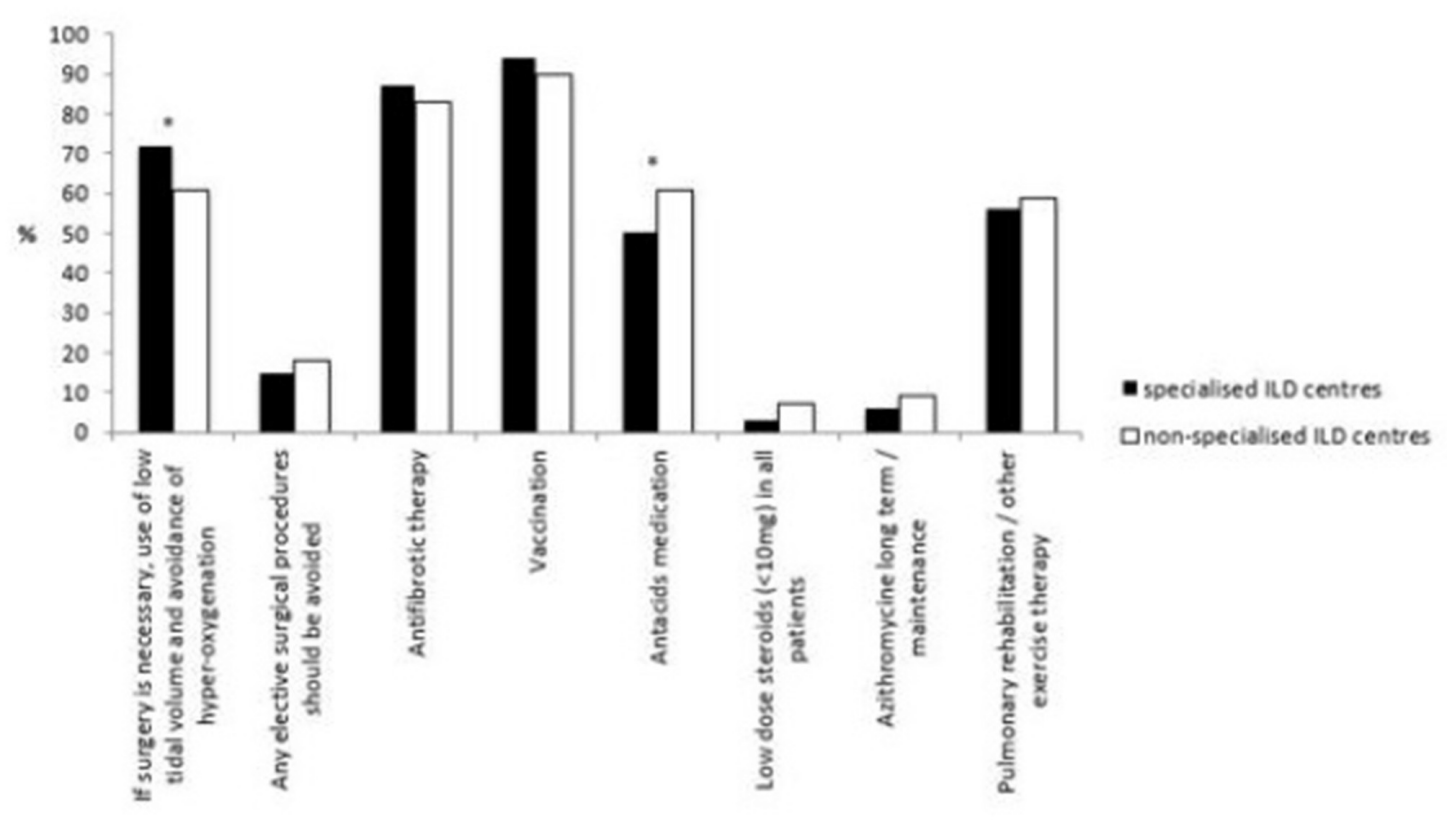
Different preventive strategies in AE-IPF in specialised and non-specialised ILD centres. Statistically significant differences are labelled with a ^*^ (p-value = <0.05).

Institutional differences in preventive strategies are shown in Figure 6. *See also* Supplementary 1.

### Unmet Needs in AE-IPF

Pulmonologists in both specialised and non-specialised ILD centres advocate more intensive collaboration between different ILD specialists; improved education and training of physicians; education of patients and caregivers as well as enhanced research to improve the understanding of the pathophysiology, diagnosis and management of AE-IPF. Physicians working in specialised ILD centres saw a stronger need for intensified research and projects on the treatment of AE-IPF (90 vs. 80%, *p* = 0.0101). There were more pulmonologists in non-specialised ILD centres who saw a need of improvement in multidisciplinary strategies for diagnosing and discussions than in specialised ILD centres (67 vs. 53%, *p* = 0.0088).

## Discussion

Despite the fact that AE-IPF is one of the most common causes of death in IPF (3), evidence on prevention, additional diagnostic approaches besides HRCT and treatment of this complication is sparse and differs significantly worldwide (10). No particular evidence-based guidance exists. Here we report analyses on similarities and differences in the management of AE-IPF in specialised vs. non-specialised ILD centres. The strength of our report was the significant number of physicians who replied to our survey representing both specialised and non-specialised ILD centres.

Diagnostic procedures were almost identical in both specialised and non-specialised ILD centres, including radiology and screening for infections. Molyneaux et al. have showed that there is an increased bacterial load in the BAL of IPF patients with AE-IPF compared to stable IPF patients (12) suggesting a potential causative role in AE-IPF. There is evidence for the contribution of lung microbiota in disease progression and in acute exacerbation (13, 14). Microbiological confirmation may therefore play an important role in the diagnostic process and may be useful for future therapeutic and preventive strategies. The majority of physicians in specialised and non-specialised ILD centres screen for pathogens in sputum, deemed a safer method to screen for pathogens compared to bronchoscopy. A recent study supports this approach as a positive bronchoscopy only affected management in 13% of patients and resulted in a change of treatment in <5% (15). Furthermore, in the same study, a significant number of patients required intubation and transfer to the ICU with poor extubation success post bronchoscopy (15). Conversely, a study has demonstrated the feasibility and safety of performing BAL aided by NIV as a useful tool for differentiating or confirming triggered AE (16).

Furthermore, there is a similarity in therapeutic approaches. The majority of pulmonologists in specialised and non-specialised ILD centres use antibiotic therapy, namely broad-spectrum antibiotic combined with macrolide. High dose steroids are widely administered in non-specialised and specialised ILD centres in AE-IPF. High dose long-term steroid use was associated with an increased mortality in the PANTHER trial (17) and a history of previous immunosuppression before AE-IPF has a negative impact on mortality (18). Recently, a retrospective study with 82 AE-IPF patients showed that subjects receiving corticosteroids were more likely to require ICU level care and mechanical ventilation and therefore did not benefit from treatment with corticosteroids (19). However, future studies with larger cohorts are necessary to prove the deleterious effects of steroid therapy.

Other immunosuppressants and strategies are used less frequently. Very few pulmonologists never use immunosuppression for AE-IPF. Although there is only low evidence base for the use of more potent anti-inflammatory treatment approaches such as cyclosporine A, intravenous cyclophosphamide or tacrolimus (20–24), they are used by some pulmonologists in non-specialised and specialised ILD centres. A randomised, double-blinded clinical trial of cyclophosphamide in AE-IPF with 120 patients was completed and results are eagerly awaited (https://clinicaltrials.gov/ct2/show/NCT02460588).

The international guidelines recommend avoiding ICU in patients with AE-IPF (weak recommendation) (25) because the mortality of patients with AE-IPF admitted to ICU, particularly in ventilated patients, is high (26). Some patients who do not respond to conventional oxygen therapy benefit from high flow oxygen (27). NIV may be a reasonable option for some critically ill patients (28). Trudzinski et al. showed that ECMO is only an option for patients who are suitable for LTX, as it conferred limited impact on the poor prognosis for those who were not LTX candidates (29). This might be a reason for pulmonologists in no matter what kind of institution to prefer NIV and high-flow oxygen in patients with AE-IPF. Data on this is however limited.

In non-specialised and specialised ILD centres prevention strategies towards AE-IPF were performed to the same extent. Vaccinations were most frequently used; although their use is recommended by the international guideline, there is a lack of evidence to support this recommendation (30).

The approach by physicians to utilise antifibrotic drugs as a form of preventive strategy is supported by recent data of controlled trials that suggest nintedanib may prolong the time to the first AE-IPF (31) and reduces mortality after AE-IPF (32). Pirfenidone reduces the risk for respiratory related hospitalisation in *post-hoc* analyses (33). Data proving a reduced frequency of AE-IPF with pirfenidone is sparse: In a Phase 2 trial of 107 patients Azuma et al. found a significant reduction of AE-IPF in patients using pirfenidone (34). A larger trial could not prove this (35).

While a Japanese study suggested a role for anticoagulants to prevent AE-IPF (Kubo et al.), a more recent study did not support this (36). This is in line with the results shown here: only a minority use anticoagulants to prevent AE-IPF. This is further supported by data suggesting a negative impact of anticoagulants on survival in IPF in general (37, 38).

Besides many similarities in the approach towards AE-IPF, there are also some differences in ILD specialised and non-specialised ILD centres.

PH is common in patients with IPF (39). Its prevalence at baseline means a higher risk for a subsequent AE-IPF, it is associated with a poorer overall survival but until now no specific PH treatment could show a benefit for IPF patients (40, 41). Significantly more pulmonologists in specialised ILD centres start diuretics compared to non-specialised ILD centres, and in line with the lack of benefits for specific PH treatment, most physicians do not use a specific PH treatment.

Many physicians use antacid drugs as a preventive strategy for AE-IPF, significantly more in non-specialised ILD centres. Lee et al. reported a higher pepsin level in the BAL of patients with AE-IPF compared to patients with stable disease (42) and a retrospective analysis showed a positive impact of antacid drugs on the course of IPF (43, 44). Other studies could not repeat this effect and suggested potentially higher rates of respiratory infections (45) and AE-IPF (46). The reason for the difference in the use of anti-acid drugs remains unclear, maybe because specialised ILD centres treat patients who are more ill or receive palliative treatment. Also different prescription rules in different countries or the fact that an old statement published by international societies from 2011 recommended their use (9) may be responsible.

Only a few pulmonologists use low dose steroids as a preventive strategy for AE-IPF, most of them in non-specialised ILD centres. This approach is in line with the international guideline where it is not recommended to use steroids beyond AE-IPF (17, 25). Furthermore, the use of corticosteroids has a negative effect on the outcome of IPF patients who received nintedanib (47).

Both pulmonologists in specialised and non-specialised ILD centres saw a high requirement for improved research strategies for AE-IPF. Significantly more non-specialised ILD centres saw the need for improvement in multidisciplinary strategies for diagnosing and discussion compared to pulmonologists in specialised ILD centres. Multidisciplinary team (MTD) meetings are widely used in the process of diagnostic and patient management (48) and they improve confidence in ILD diagnostics (49). MTD meetings are officially recommended (50). Arguably, MTD are more available in specialised ILD centres.

Many of the differences observed underscore the high and still unmet need for intensive research in AE-IPF. However, others might be associated with strategies applied outside current evidence. This demonstrates how important education in rare diseases and their complications is.

Our survey has some limitations: The survey was not designed for a data driven assessment of management practises but mainly to evaluate attitudes towards different aspects of AE-IPF. Participation was voluntarily and biased by involvement i.e. non-participating centres may have answered differently. Additionally, there was an imbalance between the number of specialised and non-specialised ILD institutions. Moreover, the number of ILD centres and patient numbers and thus experiences of diagnosis and management of AE-IPF may vary from country to country. Arguably, the number of IPF patients and cases of AE-IPF are higher in Japan as an example, than in other countries (51). Therefore, non-specialised ILD centres in Japan may have a higher number of patients to treat and thus greater experience than other non-specialised ILD centres elsewhere. More experienced physicians in the field of AE-IPF might have influenced the results of this questionnaire. Furthermore, the availability of treatments differs clearly between countries/continents, e.g., recombinant thrombomodelin is used almost exclusively in Asia and here by a quarter of all physicians (10). This might explain the fact that thrombomodulin is used more often in non-specialised ILD centres. The same applies for the use of KL-6 in the diagnostic process of AE-IPF. Finally, not all aspects of approaches to AE-IPF could be addressed in the questionnaire. The current COVID-19 pandemic was not part of the questionnaire because it was sent out before. This situation might have a huge impact on how AE-IPF is managed and this was not assed in the survey.

In conclusion, specialised and non-specialised ILD centres throughout the world do only differ in some aspects in the management of AE-IPF. Due to scant evidence and missing focused guidelines basic research and clinical trials have to be performed to establish optimal approaches to this deadly complication.

## Data Availability Statement

The original contributions presented in the study are included in the article/Supplementary Material, further inquiries can be directed to the corresponding author.

## Ethics Statement

Ethical review and approval was not required for the study on human participants in accordance with the local legislation and institutional requirements. Written informed consent from the participants was not required to participate in this study in accordance with the national legislation and the institutional requirements.

## Author Contributions

MP and MK contributed mainly to the conception and design of the study. MP wrote the first draft of the manuscript. JK performed the statistical analysis. All authors contributed to manuscript revision, read, and approved the submitted version.

## Conflict of Interest

The authors declare that the research was conducted in the absence of any commercial or financial relationships that could be construed as a potential conflict of interest.

## Publisher's Note

All claims expressed in this article are solely those of the authors and do not necessarily represent those of their affiliated organizations, or those of the publisher, the editors and the reviewers. Any product that may be evaluated in this article, or claim that may be made by its manufacturer, is not guaranteed or endorsed by the publisher.
