# Supplementary material for: Management of Acute Exacerbation of Idiopathic Pulmonary Fibrosis in Specialised and Non-specialised ILD Centres Around the World
Source: Front Med (Lausanne). 2021 Sep 27;8:699644. doi: 10.3389/fmed.2021.699644 (PMC8502934; doi:10.3389/fmed.2021.699644)
Supplement: Supplementary file 1 [file Data_Sheet_1.PDF]

Tab. 1: Diagnostic procedures of AE-IPF

	Specialised ILD centre	Non-ILD specialised centre	Total	pvalue
D-Dimer	65% (N=315)	65% (N=129)	65% (N=444)	1.0000
Troponins	52% (N=315)	47% (N=129)	50% (N=444)	0.3697
NT-proBNP/BNP	73% (N=315)	72% (N=129)	73% (N=444)	0.8812
KL-6	15% (N=315)	24% (N=129)	18% (N=444)	0.0313
HRCT / multislice thin-section CT (without contrast media)	76% (N=313)	78% (N=128)	77% (N=441)	0.7293
CT with contrast media (even in the absence of clinical suspicion of pulmonary embolism)	37% (N=313)	29% (N=128)	34% (N=441)	0.1440
Echocardiography	67% (N=313)	67% (N=128)	67% (N=441)	1.0000
Bronchoalveolar lavage always	4% (N=313)	9% (N=128)	5% (N=441)	0.1022
Bronchoalveolar lavage only if infection is suspected and the patient is in an appropriate condition to undergo bronchoscopy	73% (N=313)	68% (N=128)	72% (N=441)	0.2927
Sputum	86% (N=313)	81% (N=128)	85% (N=441)	0.2361
Induced sputum	12% (N=313)	22% (N=128)	15% (N=441)	0.0106
Urine	42% (N=313)	28% (N=128)	38% (N=441)	0.0068
CMV-PCR/CMV pp65Ag	39% (N=313)	39% (N=127)	39% (N=440)	1.0000
Pneumocystis jiroveci	61% (N=313)	55% (N=127)	59% (N=440)	0.3307
Influenza	77% (N=313)	73% (N=127)	76% (N=440)	0.5212
RSV	50% (N=313)	33% (N=127)	45% (N=440)	0.0024

Tab. 2: Treatments of AE-IPF

	Specialised ILD centre	Non-ILD specialised centre	Total	pvalue
Prednisolone 1 mg / kg / day, followed by slow tapering (over weeks)	30% (N=311)	35% (N=127)	32% (N=438)	0.3092
Methylprednisolone or equivalent 500 mg-1000 mg / day for 3 days, followed by slow tapering	63% (N=311)	66% (N=127)	64% (N=438)	0.5687
Methylprednisolone or equivalent 500 mg-1000 mg / day pulsed for 3 days WITHOUT any tapering	11% (N=311)	12% (N=127)	11% (N=438)	0.8444
Other prednisolone dosages	13% (N=311)	11% (N=127)	13% (N=438)	0.6455
Cyclosporine	6% (N=311)	13% (N=127)	8% (N=438)	0.0288
Cyclophosphamide i.v. bolus	19% (N=311)	17% (N=127)	18% (N=438)	0.6438
Tacrolimus	4% (N=311)	9% (N=127)	5% (N=438)	0.0705
Rituximab	4% (N=311)	3% (N=127)	4% (N=438)	0.8150
No use of any immunosuppressive therapy	4% (N=311)	2% (N=127)	3% (N=438)	0.4309
Polymyxin B Hemoperfusion (or similar)	7% (N=307)	10% (N=126)	8% (N=433)	0.5275
Recombinant Thrombomodulin	8% (N=307)	15% (N=126)	10% (N=433)	0.0342
Plasmapheresis / plasma exchange	4% (N=307)	5% (N=126)	4% (N=433)	0.8895
Broad-spectrum antibiotics combined with macrolides	56% (N=306)	58% (N=126)	56% (N=432)	0.7288
Antibiotic treatment only when there is a clinical and/or laboratory indication for a bacterial infection	21% (N=306)	25% (N=126)	22% (N=432)	0.3325
Always initiate or increase antacid drug therapy	17%(N=300)	22% (N=121)	19% (N=421)	0.2579

Tab. 3: Approach to antifibrotic treatment in AE-IPF

	Specialised ILD centre	Non-ILD specialised centre	Total	pvalue
		In pre-diagnosed IPF without antifibrotic treatment		
Antifibrotic treatment preferentially with Nintedanib	20% (N=303)	22% (N=124)	21% (N=427)	0.8636
Antifibrotic treatment preferentially with Pirfenidone	11% (N=303)	19% (N=124)	13% (N=427)	0.0488
Antifibrotic treatment without preference	35% (N=303)	28% (N=124)	33% (N=427)	0.2417
No antifibrotic treatment	34% (N=303)	31% (N=124)	33% (N=427)	0.6944
		When to start		
Immediately (Nintedanib)	43% (N=63)	11% (N=27)	33% (N=90)	0.0073
Only after stabilization of the patient (Nintedanib)	57% (N=63)	89% (N=27)	67% (N=90)	0.0073
Immediately (Pirfenidone)	48% (N=33)	30% (N=23)	41% (N=56)	0.2825
Only after stabilization of the patient (Pirfenidone)	52% (N=33)	70% (N=23)	59% (N=56)	0.2825
Immediately (either antifibrotic treatment)	25% (N=106)	36% (N=36)	27% (N=142)	0.2588
Only after stabilization of the patient (either antifibrotic treatment)	75% (N=106)	64% (N=36)	73% (N=142)	0.2588
		Preexisting antifibrotic treatment		
Continue unchanged	80% (N=300)	70% (N=121)	77% (N=421)	0.0513
Discontinued	5% (N=300)	7% (N=121)	6% (N=421)	0.5491
Continue at reduced dose	1% (N=300)	5% (N=121)	2% (N=421)	0.0301
Change to alternative antifibrotic drug	9% (N=300)	10% (N=121)	10% (N=421)	0.9990

Tab. 4: Approach to PH in AE-IPF

	Specialised ILD centre	Non-ILD specialised centre	Total	pvalue
Start diuretic therapy	54% (N=300)	41% (N=121)	51% (N=421)	0.0210
Perform a right heart catheterization	6% (N=300)	7% (N=121)	7% (N=421)	0.8449
Start PH specific treatment after established PH diagnosis	7% (N=300)	14% (N=121)	9% (N=421)	0.0494
Start PH specific treatment without a confident diagnosis	3% (N=300)	1% (N=121)	2% (N=421)	0.4185
Evaluate again after stabilization and possibly perform a right heart catheterization then	56% (N=300)	55% (N=121)	55% (N=421)	0.9195
No PH treatment during or after AE-IPF	25% (N=300)	25% (N=121)	25% (N=421)	1.0000

Tab. 5: ICU and palliative care in AE-IPF

	Specialised ILD centre	Non-ILD specialised centre	Total	pvalue
Invasive Ventilation for all IPF patients	9% (N=300)	11% (N=121)	10% (N=421)	0.7948
Invasive ventilation only to patients suitable for lung transplantation (LTX) as a bridge to LTX or very selected other patients	48% (N=300)	39% (N=121)	45% (N=421)	0.1240
ECMO only to patients suitable for LTX as a bridge to LTX	49% (N=300)	36% (N=121)	45% (N=421)	0.0287
High-flow oxygen	84% (N=300)	78% (N=121)	82% (N=421)	0.1641
Non-invasive ventilation	72% (N=300)	77% (N=121)	73% (N=421)	0.3685
Palliative care always/ usually considered	65% (N=299)	62% (N=121)	64% (N=420)	0.6073

Tab. 6: Prevention of AE-IPF

	Specialised ILD centre	Non-ILD specialised centre	Total	pvalue
If surgery is necessary, use of low tidal volume and avoidance of hyper-oxygenation to try to prevent injury	72% (N=297)	61% (N=119)	69% (N=416)	0.0368
Preferentially use regional anesthesia (over general) when possible	70% (N=297)	68% (N=119)	69% (N=416)	0.7827
Any elective surgical procedures should be avoided and only be performed in case of an emergency	15% (N=297)	18% (N=119)	16% (N=416)	0.4365
Antifibrotic therapy	87% (N=295)	83% (N=119)	86% (N=414)	0.3261
Vaccination (influenza, pneumococcal, etc.)	94% (N=295)	90% (N=119)	93% (N=414)	0.2282
Antacids medication (PPI, H2 blockers) in all IPF patients	50% (N=295)	61% (N=119)	53% (N=414)	0.0438
Low dose steroids (<10mg) in all IPF patients	3% (N=295)	7% (N=119)	4% (N=414)	0.1022
Anticoagulants in all IPF patients	2% (N=295)	2% (N=119)	2% (N=414)	1.0000
Azithromycine long term / maintenance	6% (N=295)	9% (N=119)	7% (N=414)	0.2890
Pulmonary rehabilitation or other forms of structured exercise therapy	56% (N=295)	59% (N=119)	57% (N=414)	0.6688
